# Evolutionary performance of zero-determinant strategies in multiplayer games

**DOI:** 10.1016/j.jtbi.2015.03.032

**Published:** 2015-06-07

**Authors:** Christian Hilbe, Bin Wu, Arne Traulsen, Martin A. Nowak

**Affiliations:** a Program for Evolutionary Dynamics, Harvard University, Cambridge MA 02138, USA; b Department of Mathematics, Harvard University, Cambridge MA 02138, USA; c Department of Evolutionary Theory, Max-Planck-Institute for Evolutionary Biology, August-Thienemann-Straße 2, 24306 Plön, Germany; d Department of Organismic and Evolutionary Biology, Harvard University, Cambridge MA 02138, USA

**Keywords:** Cooperation, Repeated games, Zero-determinant strategies, Evolutionary game theory, Public goods game

## Abstract

Repetition is one of the key mechanisms to maintain cooperation. In long-term relationships, in which individuals can react to their peers׳ past actions, evolution can promote cooperative strategies that would not be stable in one-shot encounters. The iterated prisoner׳s dilemma illustrates the power of repetition. Many of the key strategies for this game, such as *ALLD*, *ALLC*, Tit-for-Tat, or generous Tit-for-Tat, share a common property: players using these strategies enforce a linear relationship between their own payoff and their co-player׳s payoff. Such strategies have been termed zero-determinant (ZD). Recently, it was shown that ZD strategies also exist for multiplayer social dilemmas, and here we explore their evolutionary performance. For small group sizes, ZD strategies play a similar role as for the repeated prisoner׳s dilemma: extortionate ZD strategies are critical for the emergence of cooperation, whereas generous ZD strategies are important to maintain cooperation. In large groups, however, generous strategies tend to become unstable and selfish behaviors gain the upper hand. Our results suggest that repeated interactions alone are not sufficient to maintain large-scale cooperation. Instead, large groups require further mechanisms to sustain cooperation, such as the formation of alliances or institutions, or additional pairwise interactions between group members.

## Highlights

• We explore the evolution of direct reciprocity in groups of *n* players.
• We show why it is instructive to consider zero-determinant (ZD) strategies.
• ZD strategies include AllD, AllC, Tit-for-Tat, extortionate and generous strategies.
• In small groups, generosity allows the evolution of cooperation.
• In large groups, cooperation is unlikely to evolve.

## 1 Introduction

One of the major questions in evolutionary biology is why individuals cooperate with each other. Why are some individuals willing to pay a cost (thereby decreasing their own fitness) in order to help someone else? During the last decades, researchers have proposed several mechanisms that are able to explain why cooperation is abundant in nature (Nowak, 2006; Sigmund, 2010). One such mechanism is repetition: if I help you today, you may help me tomorrow (Trivers, 1971). Among humans, this logic of reciprocal giving has been documented in numerous behavioral experiments (e.g., Wedekind and Milinski, 1996; Keser and van Winden, 2000; Fischbacher et al., 2001; Dreber et al., 2008; Grujic et al., 2014). Moreover, it has also been suggested that direct reciprocity is at work in several other species, including vampire bats (Wilkinson, 1984), sticklebacks (Milinski, 1987), blue jays (Stephens et al., 2002), and zebra finches (St. Pierre et al., 2009). From a theoretical viewpoint, these observations lead to the question under which circumstances direct reciprocity evolves, and which strategies can be used to sustain mutual cooperation.

The main model to explore these questions is the iterated prisoner׳s dilemma, a stylized game in which two individuals repeatedly decide whether they cooperate or defect (Rapoport and Chammah, 1965; Doebeli and Hauert, 2005). The payoffs of the game are chosen such that mutual cooperation is preferred over mutual defection, but each individual is tempted to defect at the expense of the co-player. Theoretical studies have highlighted several successful strategies for this game (Axelrod and Hamilton, 1981; Molander, 1985; Kraines and Kraines, 1989; Nowak and Sigmund, 1992, 1993b). Evolution often occurs in dynamical cycles (Boyd and Lorberbaum, 1987; Nowak and Sigmund, 1993a; van Veelen et al., 2012): unconditional defectors (*ALLD*) can be invaded by reciprocal strategies like Tit-for-Tat (*TFT*), which in turn often catalyze the evolution of more cooperative strategies like generous Tit-for-Tat (*gTFT*) and unconditional cooperators (*ALLC*). Once *ALLC* is common, *ALLD* can reinvade, thereby closing the evolutionary cycle (Nowak and Sigmund, 1989; Imhof et al., 2005; Imhof and Nowak, 2010).

The above mentioned strategies for the iterated prisoner׳s dilemma share an interesting mathematical property: they enforce a linear relationship between the players׳ payoffs in an infinitely repeated game (Press and Dyson, 2012). For example, when player 1 adopts the strategy Tit-for-Tat, the players׳ payoffs *π*_*j*_ will satisfy the equation $\pi_{1}-\pi_{2}=0$, irrespective of player 2׳s strategy. Similarly, when player 1 adopts *ALLD*, payoffs will satisfy $c\pi_{1}+b\pi_{2}=0$ (where *c* and *b* denote the cost and the benefit of cooperation, respectively; this version of the prisoner׳s dilemma is sometimes called the donation game, see e.g. Sigmund, 2010). Finally, when player 1 applies *gTFT*, the enforced payoff relation becomes $\pi_{2}=b$. Strategies that enforce such linear relationships between payoffs have been called zero-determinant strategies, or ZD strategies (this name is motivated by the fact that these strategies let certain determinants vanish, see Press and Dyson, 2012). After Press and Dyson׳s discovery, several studies have explored how ZD strategies for the repeated prisoner׳s dilemma fare in an evolutionary context (Akin, 2013; Stewart and Plotkin, 2012, 2013; Hilbe et al., 2013a,b; Adami and Hintze, 2013; Szolnoki and Perc, 2014a,b; Chen and Zinger, 2014), and in behavioral experiments (Hilbe et al., 2014a).

Zero-determinant strategies are not confined to pairwise games; they also exist in the iterated public goods game (Pan et al., 2014), and in fact in any repeated social dilemma, with an arbitrary number of involved players (Hilbe et al., 2014b). In this way, it has become possible to identify the multiplayer-game analogues of the above mentioned strategies. For example, the multiplayer-version of *TFT* in a repeated public goods game is *proportional Tit-for-Tat* (*pTFT*): if *j* of the other group members cooperated in the previous round, then a *pTFT*-player cooperates with probability j/(n−1) in the next round, with *n* being the size of the group. Herein, we will explore the role of these recently discovered multiplayer ZD strategies for the evolution of cooperation.

We consider two evolutionary scenarios. First, we consider a conventional setup, in which the members of a well-mixed population are engaged in a series of repeated public goods games, and where successful strategies reproduce more often. In line with previous studies (Boyd and Richerson, 1988; Hauert and Schuster, 1997; Grujic et al., 2012), our simulations confirm that the prospects of cooperation depend on the size of the group. Small groups promote generous ZD strategies that allow for high levels of cooperation, whereas larger groups favor the emergence of selfish ZD strategies such as *ALLD*. For our second evolutionary scenario, we consider a player with a fixed ZD strategy whose co-players are allowed to adapt their strategies over time. Similar to the case of the repeated prisoner׳s dilemma (Press and Dyson, 2012; Chen and Zinger, 2014), the resulting group dynamics then depends on the applied ZD strategy of the focal player. But also here, the possibilities of a single player to generate a positive group dynamics diminishes with group size, irrespective of the strategy applied by the focal player.

Taken together, these results suggest that larger groups make it more difficult to sustain cooperation. In the discussion, we will thus argue that there are three potential mechanisms that can help individuals solving their multiplayer social dilemmas: they can either provide additional incentives on a pairwise basis (Rand et al., 2009; Rockenbach and Milinski, 2006); they can coordinate their actions and form alliances (Hilbe et al., 2014b); or they can implement central institutions which enforce mutual cooperation (Ostrom, 1990; Sigmund et al., 2010; Sasaki et al., 2012; Cressman et al., 2012; Traulsen et al., 2012; Zhang and Li, 2013; Schoenmakers et al., 2014).

## 2 Model

### 2.1 Iterated multiplayer dilemmas and memory-one strategies

In the following, we consider a group of *n* individuals, which is engaged in a repeated multiplayer dilemma. In each round of the game, players can decide whether to cooperate (*C*) or to defect (*D*). The payoffs in a given round depend on the player׳s own decision, and on the number of cooperators among the remaining group members. That is, in a round in which *j* of the other n−1 group members cooperate, the focal player receives *a*_*j*_ for cooperation, and *b*_*j*_ for defection (see also Table 1). We suppose that the multiplayer game takes the form of a social dilemma, such that payoffs satisfy the following three conditions (see also Kerr et al., 2004): (a) individuals prefer their co-players to be cooperative, $a_{j+1}\geq a_{j}$ and $b_{j+1}\geq b_{j}$ for all *j*; (b) within a mixed group, defectors outperform cooperators, $b_{j+1}>a_{j}$ for all *j*; (c) mutual cooperation is favored over mutual defection, $a_{n-1}>b_{0}$. Several well-known examples of multiplayer games satisfy these criteria, including the public goods game (see e.g. Ledyard, 1995), the volunteer׳s dilemma (Diekmann, 1985; Archetti, 2009), or the collective-risk dilemma (Milinski et al., 2008; Santos and Pacheco, 2011; Abou Chakra and Traulsen, 2014).

We assume that the multiplayer game is repeated, such that the group members face the same dilemma situation over multiple rounds. Herein, we will focus on infinitely repeated games, but the theory of ZD strategies can also be developed for games with finitely many rounds, or when future payoffs are discounted (Hilbe et al., 2014a, 2015). In repeated games, players can react on their co-players׳ previous behavior. In the simplest case, players only consider the outcome of the last round, that is, they apply a so-called memory-one strategy. Memory-one strategies consist of two parts: a rule that tells the player what to do in the first round, and a rule for what to do in all subsequent rounds, depending on the previous round׳s outcome. In infinitely repeated games, the first-round play can typically be neglected (see also Appendix A.1). In that case, memory-one strategies can be written as a vector $p=(p_{C,n-1},\ldots ,p_{C,0};p_{D,n-1},\ldots ,p_{D,0})$. The entries $p_{S,j}$ correspond to the player׳s cooperation probability in the next round, given that the player used S∈{C,D} in the previous round, and that *j* of the other group members cooperated. Using this notation, the strategy *ALLD* can be written as (0,…,0;0,…,0); the strategy *ALLC* takes the form (1,…,1;1,…,1); and the strategy proportional Tit-for-Tat is given by *pTFT*=($1,\frac{n-2}{n-1},\ldots ,\frac{1}{n-1},0;1,\frac{n-2}{n-1},\ldots ,\frac{1}{n-1},0$).

When all players in a group apply memory-one strategies, one can directly calculate the resulting payoffs for each group member, using a Markov chain approach (Nowak and Sigmund, 1993b; Hauert and Schuster, 1997). A detailed description is given in Appendix A.1. However, it is worth noting that the computation of payoffs is numerically expensive, because one needs to calculate the entries of a $2^{n}\times 2^{n}$ transition matrix (and the left eigenvector thereof). The exponential increase in computation time for large groups makes it difficult to attain evolutionary results beyond a certain group size (for example, in Hauert and Schuster, 1997, the maximum group size considered is *n*=5).

### 2.2 Zero-determinant strategies

Only recently, Press and Dyson (2012) have described a particular subclass of memory-one strategies for the repeated prisoner׳s dilemma. With these so-called ZD strategies, a player can enforce a linear relationship between her own payoff and the co-player׳s payoff. Such strategies do also exist in multiplayer social dilemmas (Hilbe et al., 2014b): a memory-one strategy p is called a ZD strategy if there are constants *l*, *s*, and ϕ≠0 such that the entries of p can be written as

(1) $$p_{C,j}=1+\phi [\left(1-s)(l-a_{j})-\frac{n-j-1}{n-1}(b_{j+1}-a_{j}\right)]p_{D,j}=\phi [\left(1-s)(l-b_{j})+\frac{j}{n-1}(b_{j}-a_{j-1}\right)].$$

By adopting such a strategy, player *i* can enforce the payoff relationship

(2) $$\pi_{-i}=s\pi_{i}+(1-s)l,$$

where *π*_*i*_ is the payoff of player *i*, and $\pi_{-i}=\sum_{j\neq i}\pi_{j}/(n-1)$ is the average payoff of *i*׳s co-players (Press and Dyson, 2012; Hilbe et al., 2014b). We call *s* the slope of the ZD strategy, as it controls how the co-players׳ payoffs *π*_−*i*_ change with the focal player׳s payoff *π*_*i*_. Moreover, we call *l* the baseline payoff: when all players adopt the same ZD strategy, then $\pi_{i}=\pi_{-i}$, and Eq. (2) implies that each player obtains the payoff $\pi_{i}=l$. The parameter *ϕ* in the definition of ZD strategies does not have a direct impact on the enforced payoff relationship (Eq. (2)). However, the value of *ϕ* determines how fast payoffs converge over the course of the game (Hilbe et al., 2014a). Thus, we call *ϕ* the convergence factor.

**Fig. 1:**
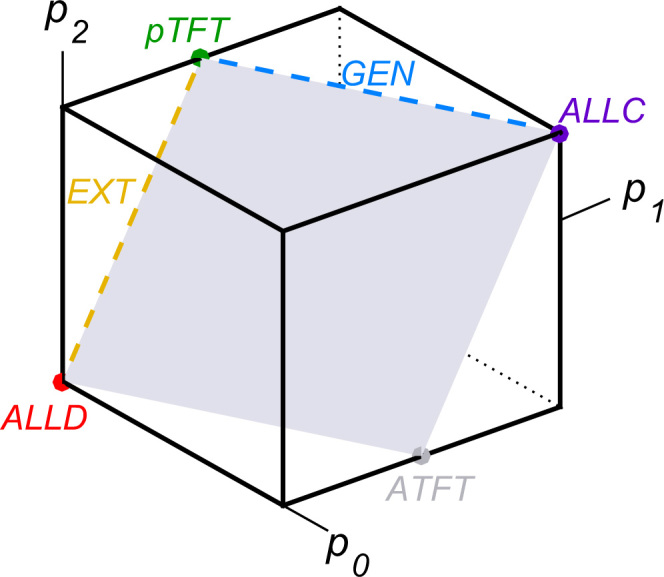
Illustration of ZD strategies for the repeated public goods game. In each panel, the focal player applies a fixed ZD strategy (*ALLD*, an extortionate strategy *EXT*, *pTFT*, a generous strategy *GEN*, or *ALLC*). The other group members are not restricted to any particular strategy. The *x*-axis depicts the resulting payoff *π*_*i*_ for the focal player, and the *y*-axis shows the corresponding average payoff of the other group members. The grey-shaded area depicts the space of all possible payoff combinations for the repeated public goods game, and the black dashed line shows the payoff combinations where the focal player yields exactly the average payoff of the other group members. The colored lines give the possible payoffs according to Eq. (2). For each ZD strategy, the parameter *s* corresponds to the slope of the colored line. Moreover, when s≠1, the parameter *l* corresponds to the intersection of the colored line with the dashed diagonal. For extortionate strategies, the line intersects the diagonal at *l*=0, and it has a positive slope s>0 (for the graph we use *s*=0.8, implying that on average, the co-players only get 80% of the extortioner׳s payoff). For generous strategies the line intersects the diagonal at the social optimum, l=rc−c, and it has a positive slope s>0. Because the colored payoff lines for *ALLD* and *EXT* are below the diagonal, this shows that defectors and extortioners earn more than average. On the other hand, *GEN* and *ALLC* yield a payoff below average. The payoff of *pTFT* always matches the average payoff of the group.

It is instructive to consider a few examples of ZD strategies for the public goods game. One example is the strategy proportional Tit-for-Tat with cooperation probabilities $p_{C,j}=p_{D,j}=j/(n-1)$. The strategy *pTFT* results from the definition of ZD strategies (1) by setting *s*=1 and ϕ=1/c. Since *s*=1, it follows from Eq. (2) that a player using *pTFT* enforces the fair relationship $\pi_{-i}=\pi_{i}$, that is, a *pTFT* player ensures that he always gets exactly the average payoff of the group. In a similar way, many well-known memory-one strategies can be represented as ZD strategies, including *ALLD*, *ALLC*, extortionate strategies (*EXT*), and generous strategies (*GEN*), as shown in Table 2 and Fig. 1.

Compared to groups with memory-one players, the calculation of payoffs becomes considerably more simple when all players adopt ZD strategies. To see this, suppose each of the *n* group members applies some ZD strategy with parameters *l*_*i*_, *s*_*i*_ and *ϕ*_*i*_. As a result, each player enforces a linear payoff relationship as in Eq. (2). Overall, this leads to *n* linear equations in the *n* unknown payoffs *π*_*i*_. This system of equations can be solved explicitly (for details, see Appendix A.2); the payoffs of the players are given by

(3) $$\pi_{i}=(1+\kappa_{i})\frac{\sum_{j=1}^{n}\kappa_{j}\cdot l_{j}}{\sum_{j=1}^{n}\kappa_{j}}-\kappa_{i}\cdot l_{i},$$

where

(4) $$\kappa_{i}≔\frac{\left(n-1)(1-s_{i}\right)}{1+(n-1)s_{i}}.$$

This formula allows a fast calculation of payoffs even in large groups.

The representation of ZD strategies in Eq. (1) has one apparent disadvantage. Because the definition requires three free parameters *l*, *s*, and *ϕ*, it may be difficult to decide whether or not a given memory-one strategy p can be written as a ZD strategy. To solve this difficulty, one can derive an alternative representation of ZD strategies. In Appendix A.3, we show that a memory-one strategy $p=(p_{S,j})$ is a ZD strategy for the public goods game if and only if the entries satisfy

(5) $$p_{S,j+1}-p_{S,j}=p_{S,j}-p_{S,j-1}\;\mathrm{for}\;S\in \{C,D\},\;1\leq j\leq n-2p_{C,j+1}-p_{D,j+1}=p_{C,j}-p_{D,j}\;\mathrm{for}\;0\leq j\leq n-2.$$

**Fig. 2:**
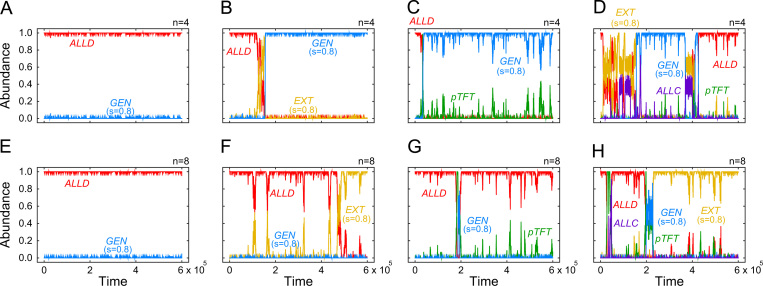
ZD strategies in the space of reactive strategies for a repeated public goods game between three players. A memory-one strategy is called reactive, if it only depends on the co-players׳ behavior, such that $p_{C,j}=p_{D,j}=p_{j}$. The space of reactive strategies is given by the cube with $0\leq p_{j}\leq 1$. The set of reactive ZD strategies is a plane connecting the points ALLD=(0,0,0), pTFT=(1,1/2,0), ALLC=(1,1,1) and the anti-reciprocal strategy ATFT=(0,1/2,1). Extortioners are on the edge with $p_{0}=0$ (extortioners never cooperate after mutual defection), whereas generous strategies are on the edge with $p_{2}=1$ (they always cooperate after mutual cooperation).

For general group sizes *n*, these conditions define the 3-dimensional subspace of ZD strategies, within the 2*n*-dimensional space of memory-one strategies. When we consider a public goods game between three players only, we can illustrate the resulting space of ZD strategies. To this end, let us assume that players only use reactive strategies (i.e., their cooperation probabilities only depend on the actions of the co-players, but not on their own action, such that $p_{C,j}=p_{D,j}≕p_{j}$ for all *j*). For groups of three players, reactive strategies thus take the form $\left(p_{2},p_{1},p_{0}\right)$, where *p*_*i*_ is the probability to cooperate when *i* of the co-players cooperated in the previous round. Since $0\leq p_{i}\leq 1$, the space of all reactive strategies takes the form of a cube (Fig. 2). The conditions for ZD strategies (5) simplify to the condition $p_{2}-p_{1}=p_{1}-p_{0}$, which is a two-dimensional plane in the cube of reactive strategies. This plane has the four corners *ALLD*, *pTFT*, *ALLC*, and the anti-reciprocal strategy ATFT=(0,1/2,1). Extortionate strategies are on the edge between *pTFT* and *ALLD*; in particular, they all have $p_{0}=0$ (extortioners never cooperate after mutual defection). In contrast, generous strategies are on the edge between *pTFT* and *ALLC*; in particular, they must have $p_{2}=1$ (generous players always cooperate after mutual cooperation).

## 3 Evolution of zero-determinant strategies

In the following, we want to explore the role of these various ZD strategies in evolutionary processes. To get an intuitive understanding of the possible transitions, let us first focus on a restricted strategy set. Specifically, we consider the strategies *ALLD*, *ALLC*, and *pTFT*; moreover, we include a particular instance of an extortionate strategy (for which we set the slope *s*=0.8, as depicted in Fig. 1B), and a particular instance of a generous strategy (also having a slope *s*=0.8, as depicted Fig. 1D). Using other instances of extortionate or generous strategies would leave the main conclusions unchanged, as described in more detail below.

For the evolutionary dynamics, we consider a population with *N* individuals. Let *N*_*D*_, *N*_*E*_, *N*_*T*_, *N*_*G*_, and *N*_*C*_ denote the number of unconditional defectors, extortioners, *pTFT* players, generous players, and unconditional cooperators, respectively, such that $N_{D}+N_{E}+N_{T}+N_{G}+N_{C}=N$. In each time step, groups of size n≤N are randomly formed (by sampling group members from the population without replacement). Given the composition of the group, we can calculate the payoff of each player using the payoff formula (3). By summing up over all possible group compositions, this yields the expected payoff π^i for each strategy i∈{D,E,T,G,C} in the population. To model the spread of successful strategies, we consider a pairwise comparison process (Blume, 1993; Szabó and Tőke, 1998; Traulsen et al., 2006; Hilbe et al., 2013a; Stewart and Plotkin, 2013). In each time step, some randomly chosen player is given the chance to imitate the strategy of some other randomly chosen group member. If the focal player׳s expected payoff is π^, and the role model׳s payoff is π^′, then the focal player adopts the role model׳s strategy with probability

(6) ρ=11+exp[−β(π^′−π^)].

The parameter β≥0 denotes the strength of selection. In the limit β→0, selection is neutral and the imitation probability simplifies to ρ=1/2. In the limit of strong selection (β→∞) the role model is imitated only if its strategy is sufficiently beneficial. In addition to these imitation events, we assume that subjects sometimes explore new strategies: in each time step, a randomly chosen player may switch to another strategy with probability μ>0 (with all other strategies having the same chance to be chosen). Overall, these assumptions lead to a stochastic selection-mutation process, in which successful strategies have a higher chance to be adopted (Nowak et al., 2004; Imhof and Nowak, 2006; Antal et al., 2009).

**Fig. 3:**
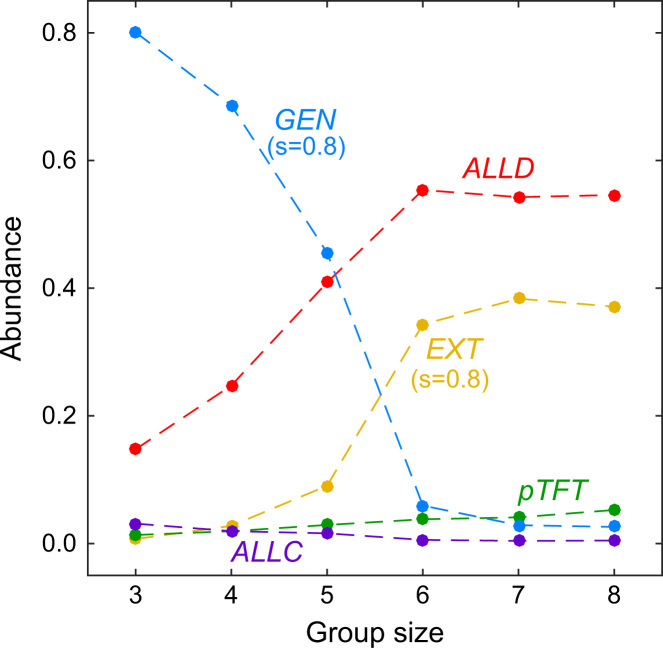
Evolution of different ZD strategies for two possible group sizes (*n*=4 or *n*=8) in a pairwise comparison process. Each panel shows the outcome of a representative simulation, starting from a homogeneous population of defectors. For the upper panels with small group sizes, we observe the following dynamics: (A) when players are only allowed to choose between *ALLD* and the generous strategy, then generous players cannot invade. (B) Adding the extortionate strategy allows generous players to take over the population. (C) Similarly, also *pTFT* can act as a catalyst for the emergence of generous strategies. (D) When all five strategies are present, cycles between cooperation and defection occur. (E)–(H) The generous strategy ceases to be stable when the groups become too large. In that case, only defectors and extortioners can spread in the population. Parameters: for the social dilemma, we have used a public goods game with *c*=1 and *r*=2; evolutionary parameters were set to β=10 and μ=0.001, and population size *N*=100. For the players׳ strategies we used the parameters in Table 2; for *EXT* and *GEN* we have set *s*=0.8 and ϕ=1/c.

To explore the role of different strategies for the evolutionary dynamics, we have run simulations for different subsets of ZD strategies, and for two different group sizes (as shown in Fig. 3). When groups are small and the population consists only of defectors and generous players, cooperation cannot emerge when initially rare (Fig. 3A). Instead, the emergence of cooperation is dependent on additional strategies that are able to invade *ALLD*. For example, extortionate strategies can serve as a catalyst for cooperation: extortioners are able to subvert defectors, and once the fraction of extortioners has surpassed a certain threshold, generous ZD strategies can invade and fixate in the population (Fig. 3B). A similar effect can be observed by adding *pTFT* to the population, which also promotes the evolution of generosity (Fig. 3C). Compared to *pTFT*, generous ZD strategies have the advantage that they are less prone to errors, as they are more likely to accept a co-player׳s accidental defection. Adding unconditional cooperators, however, can destabilize populations of generous players (Fig. 3D). *ALLC* players are able to subvert a generous population by neutral drift, which in turn allows for the re-invasion of defectors. As in the case of the iterated prisoner׳s dilemma, the dynamics of the repeated public goods game may result in cycles between cooperation and defection.

**Fig. 4:**
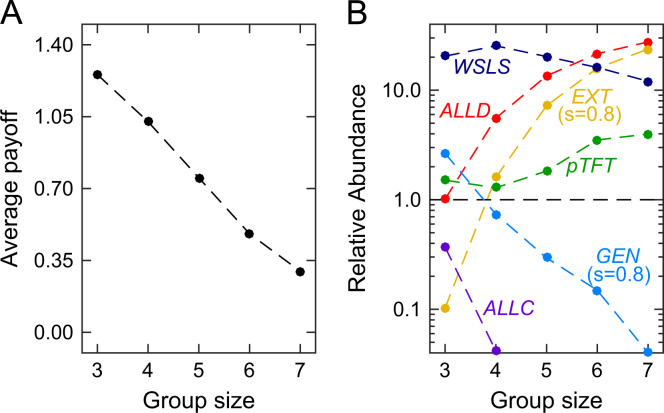
The effect of group size on the evolution of ZD strategies. We have simulated the evolutionary process when all five ZD strategies are present in the population, and calculated the average abundance of each strategy over 10^7^ time steps. Generous players are most abundant for small population sizes, whereas larger populations favor the evolution of *ALLD* and extortionate strategies. Parameters are the same as in Figure 3.

Larger group sizes further impede the evolution of cooperation: when the group size is above a certain threshold, evolution either settles at a population of defectors, or at a population of extortioners (the lower panels in Fig. 3 depict the case *n*=8). This effect of group size is also illustrated in Fig. 4, which shows the average abundance of each of the five considered ZD strategies as a function of group size *n*. Whereas generous strategies are most abundant when n<6, more selfish strategies succeed in large groups.

To obtain an analytical understanding for these results, let us calculate under which conditions a mutant ZD strategy can invade into a population of defectors. If the mutant applies a ZD strategy with parameters l^ and s^, we can use the payoff equation (3) to calculate the mutant׳s payoff in a group of defectors

(7) π^=(1−nr+(n−r)s^)l^.

Because baseline payoffs satisfy 0≤l^≤rc−c, and because slopes fulfill −1/(n−1)≤s^≤1 (Hilbe et al., 2014b), it follows that π^≤0, i.e., no single mutant has a selective advantage in an *ALLD* population. In particular, for generous mutants (with l^=rc−c and 0<s^<1) we get π^<0, and hence they are disfavored when rare. However, two strategy classes are able to invade *ALLD* by neutral drift: when the mutant either applies an extortionate strategy (with l^=0), or *pTFT* (with s^=1), then π^=0. These calculations confirm that both *pTFT* and extortionate strategies can act as a catalyst for cooperation, as they are able to subvert a population of defectors irrespective of the size of the group.

Similarly, we can also explore the stability of a population of generous players. As expected, *ALLC* mutants are always able to invade by neutral drift (again irrespective of group size). Moreover, using Eq. (3), it follows that the payoff of a single defector exceeds the residents׳ payoff rc−c if

(8) $$n>\frac{2-s}{1-s},$$

where 0<s<1 is the slope of the generous strategy. Thus, any given generous strategy can be invaded by *ALLD*, provided that the group size *n* is sufficiently large. Equivalently, to be stable against defectors, a generous strategy must not be too generous, $s>1-\frac{1}{n-1}$. In particular, it follows that the set of stable generous strategies shrinks with the size of the group. Taken together, these results suggest that it becomes increasingly difficult to achieve cooperation in large groups.

## 4 Evolution in the space of memory-one strategies

By focusing on the five ZD strategies above, we have gained insights into the possible transitions from defection to cooperation; moreover, it has allowed us to show how overly altruistic strategies (such as *ALLC*) and large group sizes can lead to the downfall of cooperation. However, the focus on these five particular strategies also comes with a risk. We may have neglected other important strategies, which may have a critical effect on the evolutionary outcomes. In order to assess how general the above results are, let us explore in the following how the dynamics of repeated social dilemmas change when we allow for all possible memory-one strategies.

Specifically, we apply the adaptive dynamics approach introduced by Imhof and Nowak (2010); that is, we adapt the previously used evolutionary process as follows. Again, we consider a population of size *N* that is engaged in a repeated public goods game, starting with a homogeneous population of defectors. When a mutation occurs, the mutant strategy is not restricted to a particular subset of ZD strategies; instead, mutants may adopt any memory-one strategy p (i.e., a mutant׳s memory-one strategy p is created by drawing 2n random numbers uniformly from the unit interval [0,1]). We assume that mutations are sufficiently rare, such that the mutant strategy either fixates, or goes extinct, before the next mutation occurs (this process may take a long time,see Fudenberg and Imhof, 2006; Wu et al., 2012). As a consequence, the dynamics results in a sequence of strategies ($p^{0}$, $p^{1}$, $p^{2}$, …), where the strategy $p^{t}$ is the strategy applied by the resident after *t* mutation events. Given this strategy sequence, we can calculate the sequence of resident payoffs (*π*^0^, *π*^1^, *π*^2^,…), using the payoff algorithm described in Appendix A.1. By analyzing these two sequences for different parameter values *n*, we can analyze the impact of group size on the evolution of strategies, and on the resulting average payoffs.

**Fig. 5:**
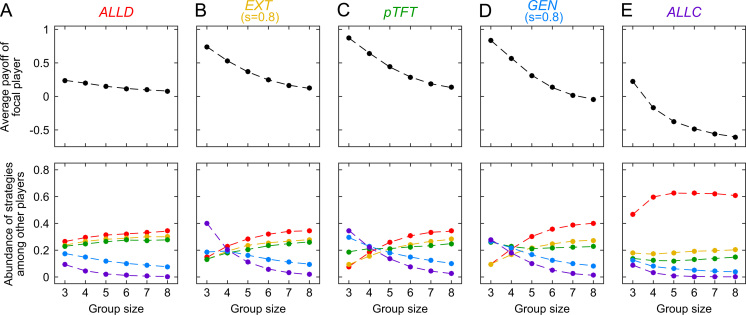
The effect of group size on average payoffs and on the relative abundance of different ZD strategies and win-stay lose-shift. Both graphs show the result of the evolutionary imitation process, in which mutant strategies either reach fixation or go extinct before the next mutant arises. The process was run for 10^7^ mutant strategies. (A) Whereas average payoffs are close to the optimum rc−c=1.4 for small group sizes, payoffs drop in large groups. (B) A strategy is favored by selection if its relative abundance is above the dashed line. For small group sizes, selection favors cooperative strategies like *WSLS* and *GEN*. As groups become larger, *EXT* and *ALLD* take over and cooperation breaks down. Parameters: efficiency *r*=2.4, cost of cooperation *c*=1, population size *N*=100, strength of selection β=1.

As shown in Fig. 5A, larger group sizes lead, on average, to lower population payoffs. This is not only in line with our previous results depicted in Fig. 4; it also confirms the results of Boyd (1989), showing that large groups are more likely to end up in selfish states. However, it is worth noting that the previous results were based on the comparison of the *ALLD* strategy with a handful of other, more cooperative strategies (in Boyd, 1989, defectors were matched against threshold variants of Tit-for-Tat,which only cooperate if at least *k* of the other players cooperated in the previous round). Fig. 5A shows that this conclusion also holds in the larger (and more general) strategy space of memory-one strategies: larger group sizes impede the evolution of cooperation (which is in line with the simulations presented in Hauert and Schuster, 1997).

To gain further insights into what drives this downfall of cooperation, we have also explored which strategies were used by the residents over the course of the evolutionary process. To this end, we have applied the method introduced by Hilbe et al. (2013a): to measure the relative importance of a given strategy p^, we have recorded how often the evolutionary process visits the neighborhood of p^ (as the strategy׳s neighborhood, we have taken the 1% of memory-one strategies that are closest to p^). Using this method, we call p^ being favored by selection if the evolutionary process spends more than 1% of the time in this neighborhood (i.e., if the process spends more time in the neighborhood than expected under neutrality).

Let us first apply this method to the five ZD strategies considered before. As shown in Fig. 5B, our results reflect the qualitative findings in the previous section. Only in small groups, the generous strategy is favored by selection; as the group size increases, *ALLD* and the extortionate strategy become increasingly successful. For comparison, we have also explored the evolutionary success of the traditional champion in repeated games, win-stay lose-shift (*WSLS*, see Nowak and Sigmund, 1993b). *WSLS* only cooperates if all group members have used the same action in the previous round, i.e., $p_{C,n-1}=p_{D,0}=1$, and $p_{S,j}=0$ otherwise (in Hauert and Schuster, 1997 this strategy is called Pavlov, and in Pinheiro et al., 2014 it is called an All-or-None strategy). In Hilbe et al. (2014b) it is shown that *WSLS* is a Nash equilibrium if $r\geq \frac{2n}{n+1}$, which is satisfied for the parameters used for the simulations. Indeed, Fig. 5B confirms that *WSLS* is favored by selection for all considered group sizes, but its relative importance decreases with *n*. For n<5, the process spends more than 20% of the time in the neighborhood of *WSLS*, whereas for *n*=7 the neighborhood is only visited 12% of the time. These results indicate that although *WSLS* is able to sustain cooperation even in larger groups, evolutionary processes tend to favor *ALLD* and extortionate strategies instead, which is in line with the downfall of average payoffs as the group size increases.

## 5 Performance of ZD strategies against adapting opponents

In the previous two sections, we have considered a traditional setup to study evolutionary processes. We have assumed that all players come from the same population, and they all are equally likely to change their strategies over time. However, for the iterated prisoner׳s dilemma it has been suggested that extortioners, and more generally ZD strategies with a positive slope, are particularly successful when they are stubborn (Press and Dyson, 2012; Hilbe et al., 2013a; Chen and Zinger, 2014): they should refrain from switching to other strategies that may be more profitable in the short run, in order to gain a long-run advantage. When a player with a fixed strategy is paired with adapting co-players, the nature of the interaction changes. Instead of a symmetric and simultaneous game, the interaction now takes the form of an asymmetric and sequential game (Bergstrom and Lachmann, 2003; Damore and Gore, 2011): by choosing a fixed strategy, the stubborn player moves first, whereas the adapting players have a chance to evolve, and to move towards a best reply over time.

To investigate such a setup in the context of multiplayer dilemmas, let us modify the evolutionary process as follows: instead of considering a large population of players, let us consider a fixed group of size *n* that is engaged in a sequence of repeated public goods game. One of the players, called the focal player, is assumed to take a fixed ZD strategy. The other group members are allowed to change their strategies from one repeated game to the next. Specifically, we assume that in each time step, one of the adapting players is chosen at random. This player is then given the chance to experiment with a different strategy. When the payoff of the old strategy is π^, whereas the new strategy yields π^′, we assume that the player switches to the new strategy with probability *ρ* as specified in Eq. (6). Overall, these assumptions result in an evolutionary process in which one player sticks to his strategy, whereas the other players can change to better strategies, given the current composition of the group.

**Fig. 6:**
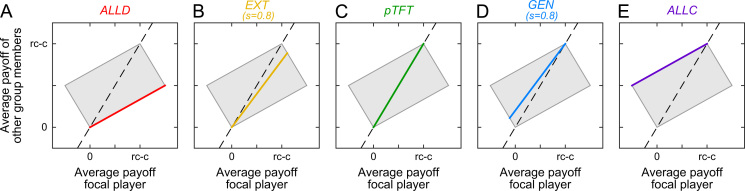
Asymmetric evolution for a focal player using a fixed ZD strategy, whereas the other group members may switch between different ZD strategies. We consider five different cases, depending on whether the focal player applies (A) *ALLD*, (B) an extortionate strategy, (C) *pTFT*, (D) a generous strategy, and (E) *ALLC*. The upper panel shows the resulting average payoff for the focal player, whereas the lower panels give the strategy abundance of the other group members, averaged over a simulation with 10^5^ adaptation events. Independent of the focal player׳s strategy, payoffs decrease with group size. However, focal players with a conditionally cooperative strategy (as in B–D) tend to have higher payoffs than unconditional focal players (as in A and E). The remaining parameters are the same as in Fig. 3: for the public goods game we have used *c*=1 and *r*=2; evolutionary parameters were set to β=10 (strong selection) and μ=0.001 (rare mutations).

In Fig. 6, we show the outcome of such an evolutionary process under the assumption that all players are restricted to the five ZD strategies used before. Independent of the fixed strategy of the focal player, all simulations have in common that the focal player׳s payoff decreases with group size. Nevertheless, the strategy of the focal player still has a considerable impact on the resulting group dynamics. For small group sizes, the simulations confirm that focal players with a higher slope value *s* tend to gain higher payoffs (see Fig. 6, upper panels). The co-players of a focal *ALLD* or *ALLC* player often adapt towards selfish strategies, whereas the co-players of a focal *EXT*, *pTFT*, or *GEN* player tend to adopt cooperative strategies (as depicted in Fig. 6, lower panels). Only as the group size becomes large, this positive effect of the focal player׳s strategy on the group dynamics disappears. For example, in groups of size *n*=8, the strategy distribution of the remaining group members is largely independent of the fixed strategy of the focal individual. The only exception occurs when the focal player is unconditionally altruistic (in which the remaining group members favor *ALLD*, independent of the group size). These simulations confirm that stubborn players are most successful when they apply ZD strategies with a high slope value (the most successful strategy in Fig. 6 is *pTFT*, which is also the strategy that has the maximum value for *s*). Higher slope values correspond to players that are more conditionally cooperative. Thus, when players aim to have a positive impact on the group dynamics, they need to apply reciprocal strategies.

## 6 Discussion

Repeated interactions provide an important explanation for the evolution of cooperation: individuals cooperate because they can expect to be rewarded in future (Trivers, 1971; Axelrod and Hamilton, 1981; Doebeli and Hauert, 2005; Nowak, 2006; Sigmund, 2010). The framework of repeated games does not necessarily require sophisticated mental capacities. Several experiments suggest that various animal species are able to use reciprocal strategies (Wilkinson, 1984; Milinski, 1987; Stephens et al., 2002; St. Pierre et al., 2009), and also theory suggests that full cooperation can already be achieved using simple strategies that only refer to the outcome of the last round.

Much research in the past has been devoted to explore conditionally cooperative strategies in pairwise interactions. There has been considerably less effort to understand the evolution of reciprocity in larger groups (some exceptions include Boyd, 1989; Hauert and Schuster, 1997; Kurokawa and Ihara, 2009; Grujic et al., 2012; Van Segbroeck et al., 2012). This is surprising, because using the theory of ZD strategies, most of the successful strategies for the repeated prisoner׳s dilemma can be naturally generalized to other social dilemmas, with an arbitrary number of players (Hilbe et al., 2014b; Pan et al., 2014). In the public goods game, for example, the set of ZD strategies includes *ALLD* and *ALLC*, but also reciprocal strategies like proportional Tit-for-Tat (*pTFT*), extortionate and generous strategies. Herein, we have explored how these strategies fare from an evolutionary perspective.

Our simulations suggest that the evolutionary success of ZD strategies critically depends on the size of the group. In smaller groups, the dynamics of strategies is comparable to the dynamics in the prisoner׳s dilemma (Nowak and Sigmund, 1992; Imhof and Nowak, 2010; Hilbe et al., 2013a): selfish populations can be invaded by extortioners or *pTFT*, which in turn can give rise to the evolution of generous ZD strategies. Generous strategies, however, can be subverted by unconditional cooperators, which can lead back to populations of defectors. These evolutionary cycles collapse when groups become too large. In large groups, evolution favors selfish strategies instead, resulting in a sharp decrease in population payoffs. To obtain these results, we have sometimes restricted the strategy space, by focusing on players using ZD strategies only. This focus has allowed us to calculate payoffs efficiently. In general, the time to compute payoffs in multiplayer games increases exponentially in the size of the group (which makes it unfeasible to simulate games with more than 5–10 players). But for ZD strategies, payoffs can be computed directly, using the formula in Eq. (3). The focus on ZD strategies, however, may come at the risk of neglecting other important strategies, such as win-stay lose-shift (*WSLS*). Nevertheless, our main qualitative results remain unchanged even when we consider the more general space of memory-one strategies (as shown in Fig. 5).

Overall, we have observed that repeated interactions can only help sustaining cooperation when groups are sufficiently small. The downfall of cooperation in large groups can be prevented if large-scale endeavors have an efficiency advantage: Pinheiro et al. (2014) observe that *WSLS* remains successful if r/n is kept constant (and therefore *r* needs to increase as *n* becomes large). However, for many examples (such as the management of common resources) such an efficiency advantage seems unfeasible. For such cases, our results suggest that repeated interactions alone are no longer able to sustain cooperation.

Yet, human societies are remarkably successful in maintaining cooperative norms even in groups of considerable size (Fehr and Fischbacher, 2003), suggesting that large-scale cooperation is based on additional mechanisms. Three mechanisms seem to be especially relevant: individuals can maintain cooperation if there are additional pairwise incentives to cooperate (Rand et al., 2009; Rockenbach and Milinski, 2006); they can increase their strategic power by coordinating their actions and by forming alliances (Hilbe et al., 2014b); or they can implement central institutions that enforce mutual cooperation (Sigmund et al., 2010, 2011; Sasaki et al., 2012; Hilbe et al., 2014b; Schoenmakers et al., 2014). Interestingly, each of these additional mechanisms is costly, and thus requires an evolutionary explanation on its own. In particular, these additional mechanisms are only likely to evolve when other, more efficient ways to establish cooperation fail. Herein, we have shown such a failure to establish cooperation when repeated interactions take place in large groups.

## Figures and Tables

**Table 1:** Payoff table for multiplayer games with *n* group members (see also Gokhale and Traulsen, 2010; van Veelen and Nowak, 2012; Gokhale and Traulsen, 2014; Peña et al., 2014; Du et al., 2014). The payoff of a player depends on the player׳s own action, and on the number of cooperating co-players. As an example of a multiplayer dilemma, we will discuss linear public good games. There, cooperators contribute an amount c>0 to a common pool. Total contributions to the common pool are multiplied by a factor *r* with 1<r<n, and evenly shared among all group members. Thus, the payoff of a cooperator is $a_{j}=\mathrm{rc}(j+1)/n-c$, whereas the payoff of a defector is $b_{j}=\mathrm{rcj}/n$.

Number of cooperating co-players	*n*−1	*n*−2	…	0
Payoff for cooperation	$a_{n-1}$	$a_{n-2}$	…	$a_{0}$
Payoff for defection	$b_{n-1}$	$b_{n-2}$	…	$b_{0}$

**Table 2:** Examples of ZD strategies for the repeated public goods game. The three strategies *ALLD*, *pTFT*, and *ALLC* can be written as ZD strategies as specified in the table. Moreover, one can define two important sub-classes of ZD strategies. Extortionate strategies (*EXT*) choose the lowest possible baseline payoff, *l*=0, and a positive slope value, 0<s<1. In this way, extortionate players ensure that their payoff is always above average, $\pi_{i}\geq \pi_{-i}$ (Hilbe et al., 2014b). Generous ZD strategies (*GEN*), on the other hand, choose the highest possible baseline payoff l=rc−c, and a positive slope value 0<s<1. As a consequence, generous players ensure that they never outperform their co-players, $\pi_{i}\leq \pi_{-i}$. An illustration of these strategies is given in Fig. 1.

Strategy	Baseline payoff *l*	Slope *s*	Convergence factor *ϕ*
ALLD	0	$\frac{(n-1)r-n}{(n-1)r}$	$\frac{(n-1)r}{\mathrm{cn}(r-1)}$
EXT	0	s>0	*ϕ*
pTFT	$\frac{\mathrm{rc}-c}{2}$	1	$\frac{1}{c}$
GEN	*rc*− *c*	s>0	*ϕ*
ALLC	*rc*− *c*	$\frac{(n-1)r-n}{(n-1)r}$	$\frac{(n-1)r}{\mathrm{cn}(r-1)}$

## Appendix A

### A.1 Payoffs in groups of memory-one players

In the following, we describe how one can calculate the average payoffs in a group, under the assumption that all players use memory-one strategies. To this end, consider a group of size *n* and suppose player *i* applies the memory-one strategy $p^{i}=(p_{C,n-1}^{i},\ldots ,p_{D,0}^{i})$. To calculate payoffs, we use the Markov-chain approach presented in Hauert and Schuster (1997). The states of the Markov chain are the possible outcomes of a given round: if the action of player *i* in a given round is *S*_*i*_, then we can write the outcome of that round as a vector $\sigma =(S_{1},\ldots ,S_{n})\in {\left\{C,D\right\}}^{n}$. Let |σ| denote the number of cooperators in *σ*. Given each player׳s memory-one strategy $p^{i}$, and the outcome *σ* of the previous round, one can calculate the transition probability $m_{\sigma ,\sigma^{\prime }}$ to observe the outcome $\sigma^{\prime }=(S_{1}^{\prime },\ldots ,S_{n}^{\prime })$ in the next round. Since players act independently, $m_{\sigma ,\sigma^{\prime }}$ is a product with *n* factors, $m_{\sigma ,\sigma^{\prime }}=\prod_{i=1}^{n}q_{i}$, where

(9) $$q_{i}=\{\begin{array}{cc} p_{C,|\sigma |-1}^{i} & \mathrm{if}\;S_{i}=C,S_{i}^{\prime }=C\\ 1-p_{C,|\sigma |-1}^{i} & \mathrm{if}\;S_{i}=C,S_{i}^{\prime }=D\\ p_{D,|\sigma |}^{i} & \mathrm{if}\;S_{i}=D,S_{i}^{\prime }=C\\ 1-p_{D,|\sigma |}^{i} & \mathrm{if}\;S_{i}=D,S_{i}^{\prime }=D. \end{array}$$

The transition probabilities $m_{\sigma ,\sigma^{\prime }}$ can be collected in a stochastic transition matrix $M=(m_{\sigma ,\sigma^{\prime }})$. In most cases, this transition matrix has a unique left eigenvector $v=(v_{\sigma })$ with respect to the leading eigenvalue 1, such that v=v·M, and $\sum_{\sigma }v_{\sigma }=1$. In that case, the entries $v_{\sigma }$ give the fraction of rounds in which the players find themselves in state *σ* over the course of the game. For each of these states *σ*, we can define the resulting payoff $g_{\sigma }^{i}$ for player *i* as

(10) $$g_{\sigma }^{i}=\{\begin{array}{cc} a_{|\sigma |-1} & \mathrm{if}\;S_{i}=C\\ b_{\left|\sigma \right|} & \mathrm{if}\;S_{i}=D \end{array}$$

As a result, we can calculate the average payoff *π*^i^ of player *i* over the course of the repeated multiplayer game as

(11) $$\pi^{i}=g^{i}\cdot v=\underset{\sigma }{\sum }g_{\sigma }^{i}\cdot v_{\sigma }.$$

In a few cases, however, the invariant distribution of the transition matrix *M* may not be unique. This happens, for example, when all players apply the strategy *pTFT*, such that the payoffs critically depend on the players׳ cooperation probabilities in the initial round. To circumvent these technical difficulties, we make the assumption that players sometimes commit errors with probability *ε*. In effect, this assumption implies that instead of the intended strategy p, players use the memory-one strategy p(ε)=(1−ε)p+ε(1−p), where 1 is the corresponding vector with all entries being one. For any 0<ε<1, the resulting invariant distribution v(ε) is unique. Payoffs are then defined by considering the limit when the error rate goes to zero (see also Section 3.14 in Sigmund, 2010),

(12) $$\pi^{i}=\underset{\varepsilon \to 0}{\lim}\;g^{i}\cdot v(\varepsilon ).$$

As an example, this definition of payoffs implies that a homogeneous group of *pTFT*-players yields a payoff of (rc−c)/2. For groups in which v(0) is unique, the payoff formulas (11) and (12) give the same result.

### A.2 Payoffs in groups of ZD strategists

When all players of a group apply a ZD strategy, the calculation of payoffs becomes considerably more simple. To show this, let us consider a group of *n* players, where each of the players applies some ZD strategy with parameters *l*_*i*_ and *s*_*i*_. It follows that each of the players enforces the payoff relationship

(13) $$\pi_{-i}=s_{i}\pi_{i}+(1-s_{i})l_{i}.$$

Instead of a relationship between player *i*׳s payoff *π*_*i*_ and the average payoff of the co-players *π*_−*i*_, we can rewrite this equation such that it is a function between *π*_*i*_ and the average payoff of the group (including *i*)

(14) $$\overset{¯}{\pi }=\frac{(n-1)s_{i}+1}{n}\pi_{i}+\frac{\left(n-1)(1-s_{i}\right)}{n}l_{i},$$

with $\overset{¯}{\pi }=\sum_{j=1}^{n}\pi_{j}/n$. This confirms that player *i*׳s payoff can be calculated once the average payoff of the group $\overset{¯}{\pi }$ is known. To calculate $\overset{¯}{\pi }$, we use elementary transformations to write the relationship (14) as

(15) $$\kappa_{i}\cdot \overset{¯}{\pi }-\kappa_{i}\cdot l_{i}=\pi_{i}-\overset{¯}{\pi },$$

with

(16) $$\kappa_{i}≔\frac{\left(n-1)(1-s_{i}\right)}{1+(n-1)s_{i}}.$$

Summing up over all players 1≤i≤n then confirms that $\sum \kappa_{i}\cdot \overset{¯}{\pi }-\sum \kappa_{i}\cdot l_{i}=0$, and therefore

(17) $$\overset{¯}{\pi }=\left(\overset{n}{\underset{j=1}{\sum }}\kappa_{j}\cdot l_{j}\right)/(\overset{n}{\underset{j=1}{\sum }}\kappa_{j})$$

Substituting this result into (15) then leads to the conclusion

(18) $$\pi_{i}=(\kappa_{i}+1)\frac{\sum_{j=1}^{n}\kappa_{j}\cdot l_{j}}{\sum_{j=1}^{n}\kappa_{j}}-\kappa_{i}\cdot l_{i}.$$

This allows a direct calculation of the payoffs from the parameters *l*_*i*_ and *s*_*i*_.

### A.3 Zero-determinant strategies for the public goods game

In the public goods game, cooperators contribute an amount c>0 into a common pool. Total contributions are then multiplied by some factor 1<r<n, and equally divided among all group members. In the following, we aim to provide an alternative characterization of ZD strategies for the public goods game (which does not depend on free parameters such as *l*, *s*, and *ϕ*). Proposition 1 Characterization of ZD-strategies for the public goods game *A memory-one strategy* $p=(p_{S,j})$ *for the public goods game is a ZD strategy if and only if*

(19) $$p_{S,j+1}-p_{S,j}=p_{S,j}-p_{S,j-1}\;\mathrm{for}\;S\in \{C,D\},\;\mathrm{and}\;1\leq j\leq n-2p_{D,j+1}-p_{C,j+1}=p_{D,j}-p_{C,j}\;\mathrm{for}\;0\leq j\leq n-2.$$

Proof

(⇒) By plugging the values of $a_{j}=\frac{(j+1)\mathrm{rc}}{n}-c$ and $b_{j}=\frac{\mathrm{jrc}}{n}$ into the definition of ZD strategies (1), it follows that any ZD strategy satisfies the following two conditions:
  (20) $$p_{C,j+1}-p_{C,j}=\phi [-(1-s)\frac{\mathrm{rc}}{n}+\frac{c}{n-1}]p_{D,j+1}-p_{D,j}=\phi [-(1-s)\frac{\mathrm{rc}}{n}+\frac{c}{n-1}].$$
  Since the two terms on the right hand side coincide, it follows that the two expressions on the left hand side coincide, and thus
  (21) $$p_{D,j+1}-p_{C,j+1}=p_{D,j}-p_{C,j}$$
  for all *j*. Moreover, since the two terms on the right hand side of (20) are independent of *j*, we can also conclude that
  (22) $$p_{S,j+1}-p_{S,j}=p_{S,j}-p_{S,j-1}$$
  for S∈{C,D} and 1≤j≤n−2.
(⇐) Conversely, for a given memory-one strategy that satisfies Eq. (19), let us define
  (23) $$l=\frac{\left(r-1)c\cdot (p_{D,j}-j(p_{C,j+1}-p_{C,j})\right)}{1-(n-1)\cdot (p_{C,j+1}-p_{C,j})+(p_{D,j}-p_{C,j})}s=\frac{-(n-1)r\cdot (p_{C,j+1}-p_{C,j})+(n-\mathrm{nr}+r)\cdot (p_{D,j}-p_{C,j})+(n-\mathrm{nr}+r)}{(n-1)(n-r)\cdot (p_{C,j+1}-p_{C,j})-(n-1)r\cdot (p_{D,j}-p_{C,j})-(n-1)r}\phi =\frac{-(n-1)(n-r)\cdot (p_{C,j+1}-p_{C,j})+(n-1)r\cdot (p_{D,j}-p_{C,j})+(n-1)r}{\mathrm{cn}(r-1)}$$
  Let us first show that these parameters are independent of *j*. For *s* and *ϕ*, the independence of *j* follows immediately from conditions (5). But also the parameter *l* is independent of *j*, which can be shown by repeatedly using (5)
  (24) $$p_{D,j}-j(p_{C,j+1}-p_{C,j})=p_{D,j}-j(p_{D,j+1}-p_{D,j})=p_{D,j}-j(p_{D,j}-p_{D,j-1})=p_{D,j}-\overset{j}{\underset{k=1}{\sum }}(p_{D,k}-p_{D,k-1})=p_{D,j}-(p_{D,j}-p_{D,0})=p_{D,0}.$$
  Thus, the above parameters *l*, *s*, and *ϕ* are indeed independent of *j*. Then,
  (25) $$p_{C,j}=1+\phi [\left(1-s)(l-a_{j})-\frac{n-j-1}{n-1}(b_{j+1}-a_{j}\right)]p_{D,j}=\phi [\left(1-s)(l-b_{j})+\frac{j}{n-1}(b_{j}-a_{j-1}\right)],$$
  which can be shown by plugging the values of *l*, *s*, and *ϕ* in (23) into the right hand side of (25). Overall, (25) confirms that p is a ZD strategy. □

We note that characterization (19) does not depend on the game parameters: the set of ZD strategies is the same for all public good games (no matter what the multiplication factor *r* is). It also follows that all unconditional strategies (such as *ALLC* and *ALLD*) are ZD strategies.
